# Construction of a gene model related to the prognosis of patients with gastric cancer receiving immunotherapy and exploration of *COX7A1* gene function

**DOI:** 10.1186/s40001-024-01783-x

**Published:** 2024-03-17

**Authors:** Si-yu Wang, Yu-xin Wang, Ao Shen, Xian-qi Yang, Cheng-cai Liang, Run-jie Huang, Rui Jian, Nan An, Yu-long Xiao, Li-shuai Wang, Yin Zhao, Chuan Lin, Chang-ping Wang, Zhi-ping Yuan, Shu-qiang Yuan

**Affiliations:** 1 Department of Oncology, The First People’s Hospital of Yibin, No. 65, Wenxing Street, Cuiping District, Yibin, 644000 China; 2 The First Hospital of Jilin University, Changchun, 130000 China; 3 Department of Thoracic Surgery, National Cancer Center/National Clinical Research Center for Cancer/Cancer Hospital, Chinese Academy of Medical Sciences and Peking Union Medical College, Beijing, China; 4 Department of Gastric Surgery, State Key Laboratory of Oncology in South China, Guangdong Provincial Clinical Research Center for Cancer, Sun Yat-sen University Cancer Center, Collaborative Innovation Center for Cancer Medicine, Guangzhou, 510060 China; 5 Department of Medical Oncology, State Key Laboratory of Oncology in South China, Guangdong Provincial Clinical Research Center for Cancer, Sun Yat-sen University Cancer Center, Collaborative Innovation Center for Cancer Medicine, Guangzhou, 510060 China

**Keywords:** Gastric cancer (GC), Biomarker, Prognostic model, Immunotherapy, *COX7A1*, Gene function

## Abstract

**Background:**

GC is a highly heterogeneous tumor with different responses to immunotherapy, and the positive response depends on the unique interaction between the tumor and the tumor microenvironment (TME). However, the currently available methods for prognostic prediction are not satisfactory. Therefore, this study aims to construct a novel model that integrates relevant gene sets to predict the clinical efficacy of immunotherapy and the prognosis of GC patients based on machine learning.

**Methods:**

Seven GC datasets were collected from the Gene Expression Omnibus (GEO) database, The Cancer Genome Atlas (TCGA) database and literature sources. Based on the immunotherapy cohort, we first obtained a list of immunotherapy related genes through differential expression analysis. Then, Cox regression analysis was applied to divide these genes with prognostic significancy into protective and risky types. Then, the Single Sample Gene Set Enrichment Analysis (ssGSEA) algorithm was used to score the two categories of gene sets separately, and the scores differences between the two gene sets were used as the basis for constructing the prognostic model. Subsequently, Weighted Correlation Network Analysis (WGCNA) and Cytoscape were applied to further screen the gene sets of the constructed model, and finally *COX7A1* was selected for the exploration and prediction of the relationship between the clinical efficacy of immunotherapy for GC. The correlation between *COX7A1* and immune cell infiltration, drug sensitivity scoring, and immunohistochemical staining were performed to initially understand the potential role of *COX7A1* in the development and progression of GC. Finally, the differential expression of *COX7A1* was verified in those GC patients receiving immunotherapy.

**Results:**

First, 47 protective genes and 408 risky genes were obtained, and the ssGSEA algorithm was applied for model construction, showing good prognostic discrimination ability. In addition, the patients with high model scores showed higher TMB and MSI levels, and lower tumor heterogeneity scores. Then, it is found that the *COX7A1* expressions in GC tissues were significantly lower than those in their corresponding paracancerous tissues. Meanwhile, the patients with high *COX7A1* expression showed higher probability of cancer invasion, worse clinical efficacy of immunotherapy, worse overall survival (OS) and worse disease-free survival (DFS).

**Conclusions:**

The ssGSEA score we constructed can serve as a biomarker for GC patients and provide important guidance for individualized treatment. In addition, the *COX7A1* gene can accurately distinguish the prognosis of GC patients and predict the clinical efficacy of immunotherapy for GC patients.

**Supplementary Information:**

The online version contains supplementary material available at 10.1186/s40001-024-01783-x.

## Introduction

Gastric cancer (GC) is one of the malignant tumors with high morbidity and mortality all over the world [1]. Data from the World Health Organization showed that among the global incidence of 1 million cases [2], GC has been listed as the fifth largest tumor burden in the world [3]. Moreover, GC is mostly in the middle and advanced stage when it is diagnosed, leading to its high mortality. The Global Cancer Report 2020 revealed that more than 760,000 GC patients died each year worldwide, making it the fifth most common cause of cancer-related death [4, 5]. In the past few decades, auxiliary examination techniques (tumor markers, CT, MRI and ultrasound, etc.), surgical operation, radiotherapy, systematic chemotherapy, targeted therapy and immunotherapy have all made great progress [6], but unfortunately, the prognosis of GC patients is still very poor, especially in those with advanced stage, with the five-year survival rate less than 20% [7–10]. Some GC patients may have the similar tumor grades and the same pathological stages, but their survival outcomes may be completely different based on different gene expression characteristics [11]. In recent years, immune checkpoint inhibitors (ICIs), such as anti-CTLA-4 (cytotoxic T lymphocyte associated antigen-4) inhibitors and anti-PD-1 (programmed cell death 1)/PD-L1 (programmed cell death ligand 1) inhibitors, as well as the combined treatment with chemotherapy and immunotherapy, have made significant progress in many types of cancers [12]. At present, several ICIs have been allowed to be used for clinical treatment of GC patients with advanced stage [13, 14]. However, the overall response rate of GC patients to current ICIs is only 20–40% [15]. Therefore, identifying effective biomarkers to screen out GC patients who may receive satisfied clinical efficacy from immunotherapy is urgently needed.

Tumor mutation burden (TMB), neoantigen load (NAL), copy alternation number (CAN), mismatch repair deficiency (dMMR), microsatellite instability (MSI), tumor microenvironment (TME), especially the expression of PD-1/PD-L1 and mutation of some specific genes are considered to be predictive markers of immunotherapy in GC patients [16–18]. However, these indicators do have limitations that hinder their clinical applications, such as spatio-temporal heterogeneity, low accuracy or less applicable population [19, 20]. Therefore, in the era of individualized medical treatment, it is imperative to find reliable and accurate biomarkers to enhance the clinical efficacy of immunotherapy for GC patients.

Human *COX7A1* gene encodes cytochrome C oxidase subunit 7A1 protein [21, 22], playing a role in a multi-unit heterologous complex (such as complex IV) of the mitochondrial respiratory chain. The complex IV consists of three catalytic subunits encoded by mitochondrial genes and multiple structural subunits [23]. In recent years, some researchers have found that *COX7A1* is related to the metabolism and treatment of human cancer cells [24, 25]. *COX7A1* has been widely studied in the field of lung cancer, and it has been confirmed that *COX7A1* can block autophagy by up-regulating NOX2 and down-regulating PGC-1α. At the same time, the overexpression of *COX7A1* can inhibit the proliferation and colony formation ability of human non-small cell lung cancer cells, which also depends on the regulation of autophagy [24]. The relationship between *COX7A1* and ferroptosis has also been revealed in lung cancer. *COX7A1* can increase the sensitivity of lung cancer cells to ferroptosis induced by cysteine deprivation by promoting the activity of tricarboxylic acid cycle and complex IV in mitochondrial electron transport chain [26]. Furthermore, *COX7A1* is also a super enhancer (SE) gene [27]. Super enhancers (SEs), as a kind of cis regulatory element with super transcriptional activation, was first proposed by Richard A. Young from White Institute for Biomedical Research [28, 29]. Compared with the typical enhancer (TE), the span of the SEs region is usually 8–20 Kb, which is much higher than the 200–300 Bp span of the TEs. More importantly, SEs have higher density of transcriptional activation related histone modifications (such as H3K27ac, H3K4me1, etc.), media complexes and bromodomain containing 4 (BRD4, which binds to histone acetylation modification sites) than TEs, making them have greater regulatory potential for tumor cells. Richard A. Young has mentioned that SEs are golden targets for targeted research. And in recent years, the drug clinical experimental researches on SEs advanced continuously and have achieved satisfactory results, such as those aiming at SE gene family BRD2, BRD3 and BRD4, and those aiming at CDK7 target [30]. Therefore, the research on SEs and drug targets may be the core field of tumor therapy in the future. However, SEs of *COX7A1* have not been reported yet, so we decided to explore the potential function of *COX7A1* in GC.

**Fig. 1:**
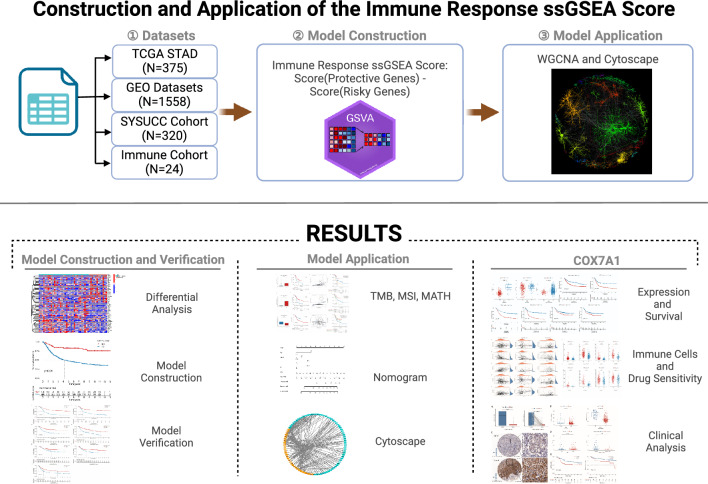
The specific workflow of our study

At present, there have been many reports on the construction of prognostic models for GC, such as those based on genes related to Notch pathway [31], those based on angiogenesis-related genes [32], and those based on apoptosis-related genes [33]. These models were reported to be able to satisfactorily distinguish GC patients and describe the clinical and molecular characteristics of these patients with different prognosis. However, these prognostic models do not further complete the screening of target molecules for potential applications. So in this study, novel modeling methods were applied to establish a complete screening strategy for exploration of target molecules related to GC immunotherapy, and the above ideas were verified by the following immunohistochemical staining. The specific workflow is shown in Fig. 1. In this study, we proposed a new process for screening genes related to the prognosis of immunotherapy, including identifying differential genes related to immunotherapy, constructing a prognostic model based on ssGSEA algorithm, and screening candidate target genes based on WGCNA and Cytoscape. At the same time, the clinical specimens of GC tissues in Sun Ya-sen University Cancer Center were acquired to further demonstrate the reliability of our screening process and the accuracy of our model, and also revealed the role of *COX7A1* as a SE in predicting the prognosis of GC patients and the clinical efficacy of immunotherapy.

## Method

### Data collection

We downloaded the transcriptome FPKM (Fragments Per Kilobase per Million) data and clinical information data of GC/STAD patients (stomach adenocarcinoma) (including age, sex, TNM stage, and follow-up data, etc.) from the official website of TCGA database (*N* = 375), and cleansed the data with Tidyverse package [34] in R software. At the same time, GC transcriptome data and clinical information of GC patients were downloaded from GEO database, such as GSE84437 (*N* = 433), GSE66229 (*N* = 300), GSE15459 (*N* = 191), GSE26253 (*N* = 432) and GSE26942 (*N* = 202). The details of the data sets can be found in Additional file 1: Table S1. We re-annotated the gene information in the GEO data sets using the sequence information GRCh38 (Genome Reference Consortium Human Build 38) obtained from the latest version of the Human Genome Project. Finally, the Kim Cohort [15] (*N* = 45) is a GC cohort that has been treated with anti-PD-1, and their transcriptome data were downloaded for reprocessing and quantifying the expression profile [35]. The baseline characteristics of GC patients in the above datasets are presented in Table 1.

**Table 1:** Baseline characteristics of GC patients from public databases in this study

Cohorts	TCGA (*N* = 375)	GSE84437 (*N* = 433)	GSE66229 (*N* = 300)	GSE15459 (*N* = 191)	GSE26253 (*N* = 432)	GSE26942 (*N* = 202)	Kim cohort (*N* = 45)
Survival status							
Alive	224 (59.7%)	224 (51.7%)	148 (49.3%)	96 (50.3%)	265 (61.3%)	114 (56.4%)	/
Dead	150 (40.0%)	209 (48.3%)	152 (50.7%)	95 (49.7%)	167 (38.7%)	88 (43.6%)	/
NA	1 (0.3%)	0 (0%)	0 (0%)	0 (0%)	0 (0%)	0 (0%)	/
Gender							
Female	134 (35.7%)	137 (31.6%)	101 (33.7%)	67 (35.1%)	152 (35.2%)	60 (29.7%)	/
Male	241 (64.3%)	296 (68.4%)	199 (66.3%)	124 (64.9%)	280 (64.8%)	142 (70.3%)	/
Age							
< 56	63 (16.8%)	144 (33.3%)	76 (25.3%)	43 (22.5%)	253 (58.6%)	90 (44.6%)	/
> = 56	308 (82.1%)	289 (66.7%)	224 (74.7%)	148 (77.5%)	179 (41.4%)	112 (55.4%)	/
NA	4 (1.1%)	0 (0%)	0 (0%)	0 (0%)	0 (0%)	0 (0%)	/
Stage							
I	53 (14.1%)	18 (4.2%)	30 (10.0%)	31 (16.2%)	68 (15.7%)	51 (25.2%)	0 (0%)
II	111 (29.6%)	206 (47.6%)	97 (32.3%)	29 (15.2%)	167 (38.7%)	36 (17.8%)	0 (0%)
III	150 (40.0%)	209 (48.2%)	96 (32.0%)	72 (37.7%)	130 (30.1%)	63 (31.3%)	0 (0%)
IV	38 (10.1%)	0 (0%)	77 (25.7%)	59 (30.9%)	67 (15.5%)	51 (25.2%)	45 (100.0%)
NA	23 (6.1%)	0 (0%)	0 (0%)	0 (0%)	0 (0%)	1 (0.5%)	0 (0%)
Lauren classification							
Diffuse	/	/	135 (45.0%)	75 (39.3%)	/	42 (20.8%)	/
Intestinal	/	/	146 (48.7%)	98 (51.3%)	/	141 (69.8%)	/
Mixed	/	/	19 (6.3%)	18 (9.4%)	/	7 (3.5%)	/
NA	/	/	0 (0%)	0 (0%)	/	12 (5.9%)	/
Stage T							
T1	19 (5.1%)	11 (2.5%)	0 (0%)	/	/	/	/
T2	80 (21.3%)	38 (8.8%)	186 (62.0%)	/	/	/	/
T3	168 (44.8%)	92 (21.2%)	91 (30.3%)	/	/	/	/
T4	100 (26.7%)	292 (67.5%)	21 (7.0%)	/	/	/	/
Tx	8 (2.1)	0 (0%)	2 (0.7%)	/	/	/	/
Stage *N*							
N0	111 (29.6%)	80 (18.5%)	38 (12.7%)	/	/	/	/
N1	97 (25.9%)	188 (43.4%)	131 (43.6%)	/	/	/	/
N2	75 (20.0%)	132 (30.5%)	80 (26.7%)	/	/	/	/
N3	74 (19.7%)	33 (7.6%)	51 (17.0%)	/	/	/	/
Nx	18 (4.8%)	0 (0%)	0 (0%)	/	/	/	/
Stage M							
M0	330 (88.0%)	433 (100.0%)	273 (91.0%)	/	/	187 (92.6%)	0 (0%)
M1	25 (6.7%)	0 (0%)	27 (9.0%)	/	/	14 (6.9%)	45 (100.0%)
Mx	20 (5.3%)	0 (0%)	0 (0%)	/	/	1 (0.5%)	0 (0%)
Chemotherapy							
Yes	/	/	/	/	432 (100.0%)	106 (52.5%)	/
No	/	/	/	/	0 (0%)	96 (47.5%)	/
Overall response (immunotherapy)							
CR	/	/	/	/	/	/	3 (6.7%)
PR	/	/	/	/	/	/	9 (20.0%)
SD	/	/	/	/	/	/	15 (33.3%)
PD	/	/	/	/	/	/	18 (40.0%)

*NA* not available, *CR* complete response, *PR* partial response, *SD* stable disease, *PD* progressive disease

### Differential gene expression analysis

In bioinformatics, differential expression analysis is a common method to study the differences of gene expression in different biological samples under different conditions. Limma package [36] is widely used for differential expression analysis, which can be used to analyze RNA-seq and microarray data, with high accuracy, reliability, flexibility and expansibility. Based on the statistical model of Bayesian method, limma package can not only control the error rate, but also find the differential expression of one or more genes between different groups, thus helping biologists to understand the mechanism of gene regulation and biological processes. Therefore, limma package [36] in R software was used to compare GC patients who had no response to immunotherapy with those who had response for obtaining differential genes, and the Benjamini–Hochberg multiple correction method was applied to calculate the FDR (false discovery rate) value of each differential gene. FDR < 0.05 and | Log_2_ FC |> 1 were set as the criteria for screening differential genes.

### Construction of gene model related to prognosis and immunotherapy

Gene set variation analysis (GSVA) provides the estimation of pathway activity by transforming the input gene expression data matrix into the corresponding gene set expression data matrix. Then the expressed data matrix can be used with classical analysis methods, such as differential expression, classification, survival analysis, clustering or correlation analysis. However, single sample GSEA (Single Sample Gene Set Enrichment Analysis, ssGSEA) is a nonparametric method, which calculates the gene enrichment score of each sample as the normalized difference of the empirical cumulative distribution function (CDF) of gene expression inside and outside the gene sets. So ssGSEA was performed to calculate enrichment scores for the above-mentioned two types of genes (HR < 1 in Cox regression analysis was defined as protective gene; HR > 1 in Cox regression analysis was defined as risky gene), and then a gene model related to the prognosis of immunotherapy was constructed by the following formula:

$${\text{ssGSEA Score }} = {\text{ Score }}\left( {\text{protective gene}} \right) \, - {\text{ Score }}\left( {\text{risky gene}} \right)$$

### Functional enrichment analysis

ClusterProfiler is a powerful bioinformatics tool that supports the use of up-to-date gene annotation data to quickly explore the functional characteristics of thousands of species, including coded and non-coded genomic data. The tool provides a general interface, which can obtain gene function annotation information from various sources and apply flexibly in different application scenarios. At the same time, Kyoto Encyclopedia of Genes and Genomes (KEGG) is an encyclopedia of genes and genomes, in which molecular functions are mainly presented as a network of interactions and responses in the form of KEGG pathways and modules. To explore the potential function of the appealing protective and risky gene sets, we performed KEGG enrichment analysis of the two gene sets based on Clusterprofiler package [37, 38], and screened out the statistically significant pathways with *P* < 0.05, so as to explore the potential biological value of the gene sets.

### Construction and evaluation of nomogram

Nomogram is widely used in cancer prognosis mainly because they can simplify statistical prediction models to a single numerical estimate of event probability, such as death or recurrence, which is tailored to individual patient conditions. After univariate Cox regression analysis, following multivariate Cox regression analysis was performed to screen all independent prognostic factors to construct a prognostic Nomogram map to evaluate the probability of 1-, 3- and 5-year overall survival (OS) of GC patients in TCGA + GSE84437 using R package “rms”. The clinical factors included age, sex, T stage, N stage and ssGSEA score. Then the calibration curve was drawn by R package “regplot”, which is mainly used to compare the probability of predicting OS with the actual OS probability of Nomogram, and to evaluate the discriminant ability of line chart graphically.

### Weighted correlation network analysis (WGCNA)

WGCNA is an algorithm based on high-throughput gene co-expression profile analysis, which is widely used to identify gene co-expression networks for various diseases. Compared with traditional methods, WGCNA is superior in analyzing gene association patterns and associating gene co-expression modules with clinical features. The construction of co-expression module based on WGCNA method includes the following main steps: first, the correlation coefficient matrix between genes was constructed, which is called adjacency matrix; then the adjacency matrix *a*_*ij*_ was used to calculate the connection strength between each pair of nodes through the following formula.

$${Z}_{ij}=\left[{\text{cor}}\left({b}_{i},{b}_{j}\right)\right]{a}_{ij}={Z}_{ij\beta }$$

Vectors (*b*_*i*_ and *b*_*j*_) are the expression values of genes, while the Pearson correlation coefficients of genes *i* and *j* and *a*_*ij*_ are expressed as the connection strength between genes. At the same time, to ensure the scale-free network in the adjacency matrix, the appropriate soft threshold power *β* = 4 is selected to ensure the scale-free topology. Then the hierarchical clustering of the weighted coefficient matrix was used to define the module. The functional modules in the co-expression network with defined genes were screened. Topology measurement (TOM) showed the concurrency of shared adjacent genes through the following formula.

$$TOMi,j=\frac{{\Sigma }^{N}{}_{K=1}{A}_{i,j}\cdot {A}_{k,j}+{A}_{i,j}}{min\left({K}_{i,}{K}_{j}\right)+1-{A}_{i,j}}$$

The *A* in the formula was the weighted adjacency matrix described in the above formula. The dissimilarity measurement based on TOM is carried out for gene tree map and average linkage hierarchical clustering, and the similar expression profile was divided into the same gene module by dynamic tree cutting package. In addition, the minimum number of genes in each gene co-expression module was set to 100, and the cutting height threshold of merging similar gene modules was set to 0.3. Finally, Pearson correlation analysis was carried out to verify the correlation between gene co-expression module and clinical parameters. Therefore, the key gene modules most significantly related to clinical parameters were identified by WGCNA algorithm for the following analysis.

### Drug sensitivity analysis

Genomics of Drug Sensitivity in Cancer (GDSC) is a common data set [39] containing drug sensitivity data (IC_50_) of 1000 cell lines to obtain information on drug sensitivity and drug resistance of GC cell lines. The OncoPredict package was developed by Maeser et al. [40] to predict the drug response of cancer patients in vivo, in which OncoPredict matched the tissue gene expression profile with the half maximal inhibitory concentration (IC_50_) of the cancer cell line, and then applied the ridge regression algorithm to predict the drug response of the samples in the TCGA + GSE84437, GSE66229, GSE26253 and GSE15459 cohort. By downloading the GDSC2 gene expression profile and corresponding drug response information using oncoPredict package, we generated a ridge regression model that can be applied to GC transcriptome data, and then obtained the sensitivity score to predict the IC_50_ of 5-Fu and Oxaliplatin.

### Immune infiltration analysis

To describe the tumor immune microenvironment, we estimated the abundance of Tumor Infiltration Immune Cell (TIIC) in each sample by CIBERSORT algorithm. CIBERSORT algorithm is an immunological calculation method based on gene expression characteristic matrix with various marked genes. It uses linear Support Vector Regression (SVR) machine learning method to deconvolution gene expression. The original gene expression data from TCGA and GEO were normalized before CIBERSORT analysis. Then, on the CIBERSORTx website (https://cibersortx.stanford.edu/), we downloaded the matrix of sample immune cell infiltration. It contains 22 immune cell subtypes of TIIC (B cells naïve, B cells memory, Plasma cells, T cells CD8, T cells CD4 naïve, T cells CD4 memory resting, T cells CD4 memory activated, T cells follicular helper, T cells regulatory (Tregs), T cells gamma delta, NK cells resting, NK cells activated, Monocytes, Macrophages M0, Macrophages M1, Macrophages M2, Dendritic cells resting, Dendritic cells activated, Mast cells resting, Mast cells activated, Eosinophils Neutrophils). At the same time, to improve the accuracy of the deconvolution algorithm, we consider the *P* value and root mean square error of CIBERSORT. The correlation between *COX7A1* expression and immune cell content was calculated by Spearman correlation test in TCGA + GSE84437, GSE66229 and GSE26253 cohorts.

### Immunohistochemistry

SYSUCC Cohort, including paraffin-embedded GC and paracancerous tissues, was collected from Sun Yat-sen University Cancer Center. At the same time, we also collected 24 GC patients to form an Immune Cohort. The specific steps of immunohistochemistry are as follows: take out a proper amount of stained slides from the refrigerator at – 80 ℃ and bake them in an oven at 60 ℃ overnight, then use xylene dewaxing and gradient alcohol hydration, and use sodium citrate buffer for antigen repair to expose antigenic determinants. Then hydrogen peroxide was used to block endogenous peroxidase and an appropriate amount of goat serum blocking solution (usually 50 ul) was used for serum blocking. After that, the *COX7A1* antibody of Affinity Biosciences was diluted and incubated at 1:50 for 1 h (37 ℃). After PBS cleaning, the HRP labeled polymer (anti-rabbit) was added to incubate the second antibody. After cleaning the glass slides by PBS, DAB solution was prepared by mixing 1ml solution B and 20 ul solution C (50:1 ratio). Then add 50–70 ul DAB solution to each slide and incubate for 1–3 min until the tissue was stained brown. Soak the slides in hematoxylin solution for 1–3 min until the nucleus was evenly stained dark blue. Then immediately put the slide into the tap water to stop dyeing. After that, the film was sealed and photographed under microscope, and the following IHC score criteria were used: positive IHC staining showed brown granules in the cytoplasm, cell membrane or nucleus. Then the dyeing intensity and dyeing area were evaluated to determine the score. Here, the staining area score was calculated as the percentage of positive staining cells in the whole tissue section, ranging from 0 to 100%. The score of staining intensity was as follows: 0 for negative staining (-); 1 for weak yellow staining; 2 for yellow staining; 3 for brown staining. The H score was calculated as the product of the percentage of dyeing area and the score of dyeing intensity, ranging from 0 to 3.

### Patients and specimens

We collected wax blocks from 320 GC patients (including 170 GC issues and 150 paracancerous tissues) and the clinical information of the corresponding patients from Sun Yat-sen University Cancer Center for external verification. These specimens did not receive any neoadjuvant therapy before operation, and all received GC surgery in Sun Yat-sen University Cancer Center from 2007 to 2010. They were followed up every 3 months in the first 2 years and every 6 months in the following 3 years. Each patient was followed up until December 31, 2015. OS was determined from the operation date to the last follow-up or death, and disease-free survival (DFS) was determined from the operation date to the last follow-up or cancer progression. At the same time, we also recruited 24 patients from Sun Yat-sen University Cancer Center to form an Immune Cohort, and the response to immunotherapy was evaluated by pathologists in our hospital according to tumor regression grade (TRG). The clinical information and the results of TRG evaluation of the 24 patients are detailed in Additional file 1: Table S2 (Immune Cohort). This study was approved by the Ethics Committee of Sun Yat-sen University Cancer Center, and all patients provided informed consent for the use of their information and samples for research purposes.

### Statistical method

All bioinformatics statistical analysis was carried out by R software (R version 4.2.2). All *P* values have passed the double-tail test, and *P* < 0.05 is considered to be statistically significant. Wilcoxon test was used to compare the difference between ssGSEA score low score group and high score group. Spearman correlation analysis was used to estimate the correlation between quantitative variables of non-normal distribution. The survival curve was evaluated by Kaplan–Meier method and Cox proportional hazard regression model, and the difference was analyzed by log rank test, in which we used two R packages [41] of “survival” and “surminer” for survival analysis and regression analysis. Before Kaplan–Meier analysis, the best grouping method was used to divide gene expression into high expression group and low expression group. Here, “ggplot2” R package [42] was used to draw the survival curves.

See “Additional file 6” for “Single-cell Data Download and Processing” and “Tumor Microenvironment Analysis”.

## Results

### Construction and verification of a gene model related to prognosis and immunotherapy

**Fig. 2:**
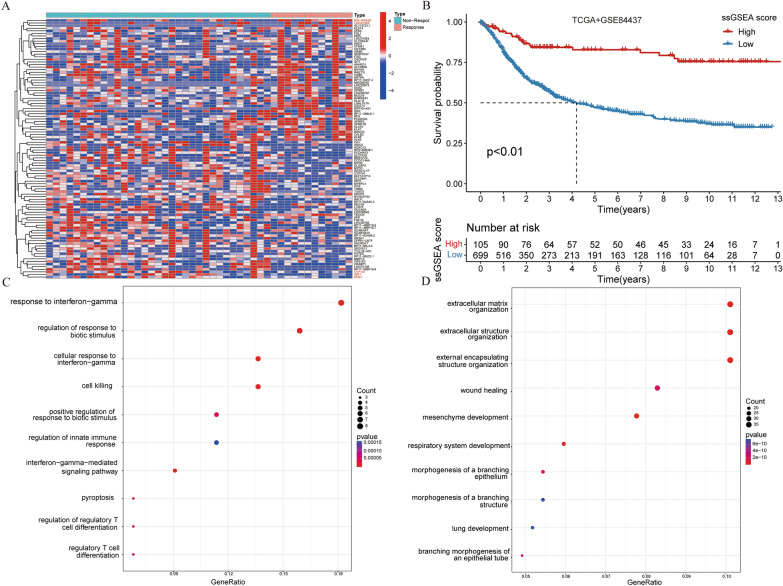
Screening of differential expression genes and construction of prognostic model for GC. **A** Differential gene heat map of response and non-response groups; **B** The survival curve of high and low risk groups of GC patients obtained by model scoring; **C**, **D** Bubble diagrams for KEGG enrichment analysis of protective and risky genes

**Fig. 3:**
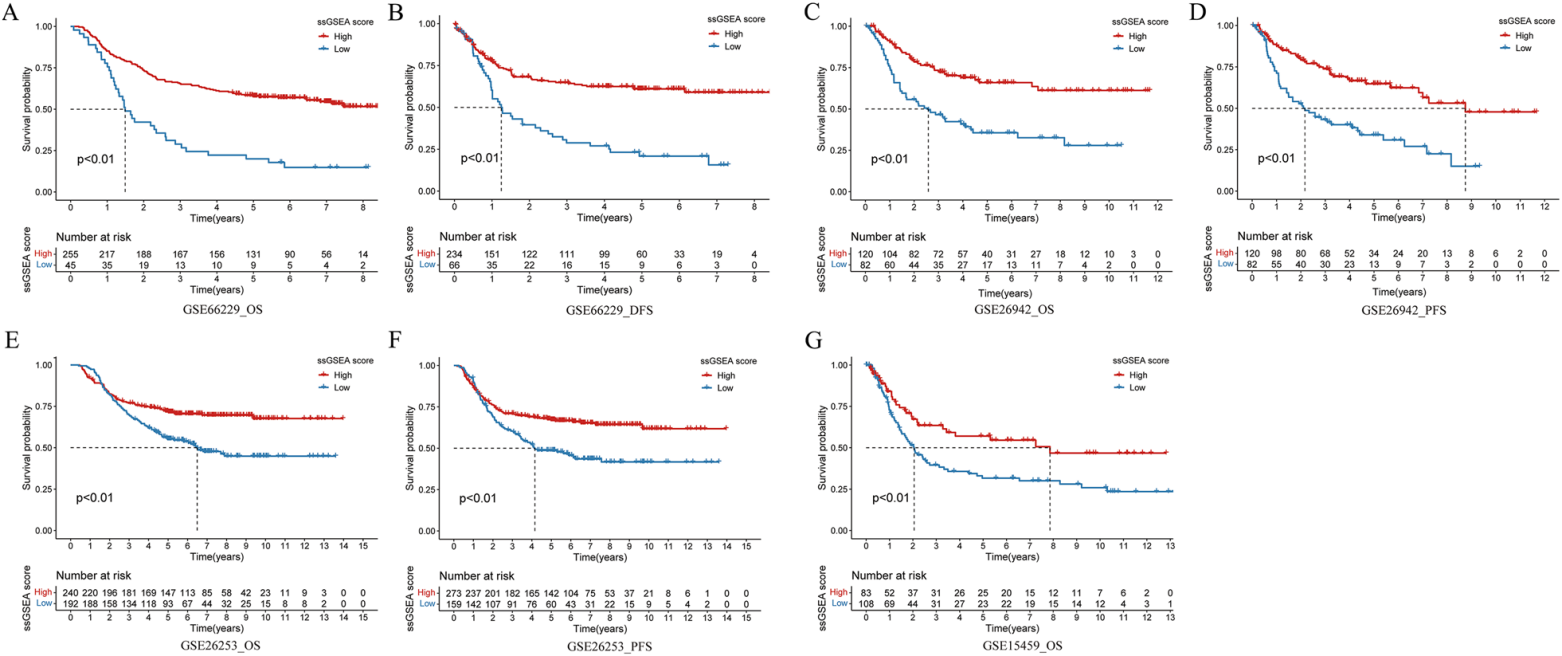
Immune response ssGSEA score can predict the OS, DFS and PFS of GC patients. **A**, **B** Survival analysis (OS and DFS) between high and low score groups of GC patients in GSE66229; **C**, **D** Survival analysis (OS and PFS) between high and low score groups of GC patients in GSE26942; **E**, **F** Survival analysis (OS and PFS) between high and low score groups of GC patients in GSE26253; **G** Survival analysis (OS) between high and low score groups of GC patients in GSE15459

The transcriptional data of Korean GC immunotherapy cohort and the clinical information of patients were analyzed by limma package (12 patients in the response group and 33 patients in the non-response group). The differential expression genes in response group and non-response group were compared (| LogFC |> 1). The differential gene heat map showed 100 genes with the most significant differences (Fig. 2A), indicating that MIA-RAB4B and LINC01433 were significantly over-expressed in the response group, while ROS1, WIF1 and CYP1A1 were significantly lower in the response group. Next, we further screened the differential expression genes between the two groups and construct a model related to the prognosis of GC. We downloaded GC dataset from TCGA and GEO, and merged them after de-batch processing. Then univariate Cox regression analysis was applied to filter 455 genes related to the prognosis. The genes with HR < 1 were defined as protective genes (47 genes), and those with HR > 1 were defined as risky genes (408 genes). The specific gene sets are shown in Additional file 3: Table S3, and then the two types of genes are analyzed by KEGG enrichment analysis. The results of enrichment analysis are also shown in Additional file 3: Table S3. Protective genes were mainly enriched in immunotherapy-related pathways such as “Response to infereron-gamma”, while risky genes were mainly enriched in immune microenvironment-related pathways such as “extracellular matrix organization” and “extracellular structure organization” (Fig. 2C, D). Then the immune response ssGSEA score was obtained by enriching the two kinds of gene sets by ssGSEA, and the gene model related to prognosis of immunotherapy was constructed. It is found that the prognosis of GC patients with high score was better than those with low score (*P* < 0.01, Fig. 2B). To verify the reliability of the model, we used GSE66229, GSE26942, GSE26253 and GSE15459 of GEO dataset for external validation. We found that the OS, DFS and progression-free survival (PFS) of GC patients with high score in GSE66229, GSE26942, GSE26253 dataset were significantly higher than those with low score (all *P* < 0.01, Fig. 3A–G), which further verified that our immune response ssGSEA score could effectively distinguish the prognosis of GC patients. We additionally assessed the prognosis at different TNM stages, and the results are shown in Additional file 5: Fig. S1. In most conditions, our model still showed an eligible predictive effect on patients at different TNM stages.

### The correlation between immune response ssGSEA score and several known markers for immunotherapy

**Fig. 4:**
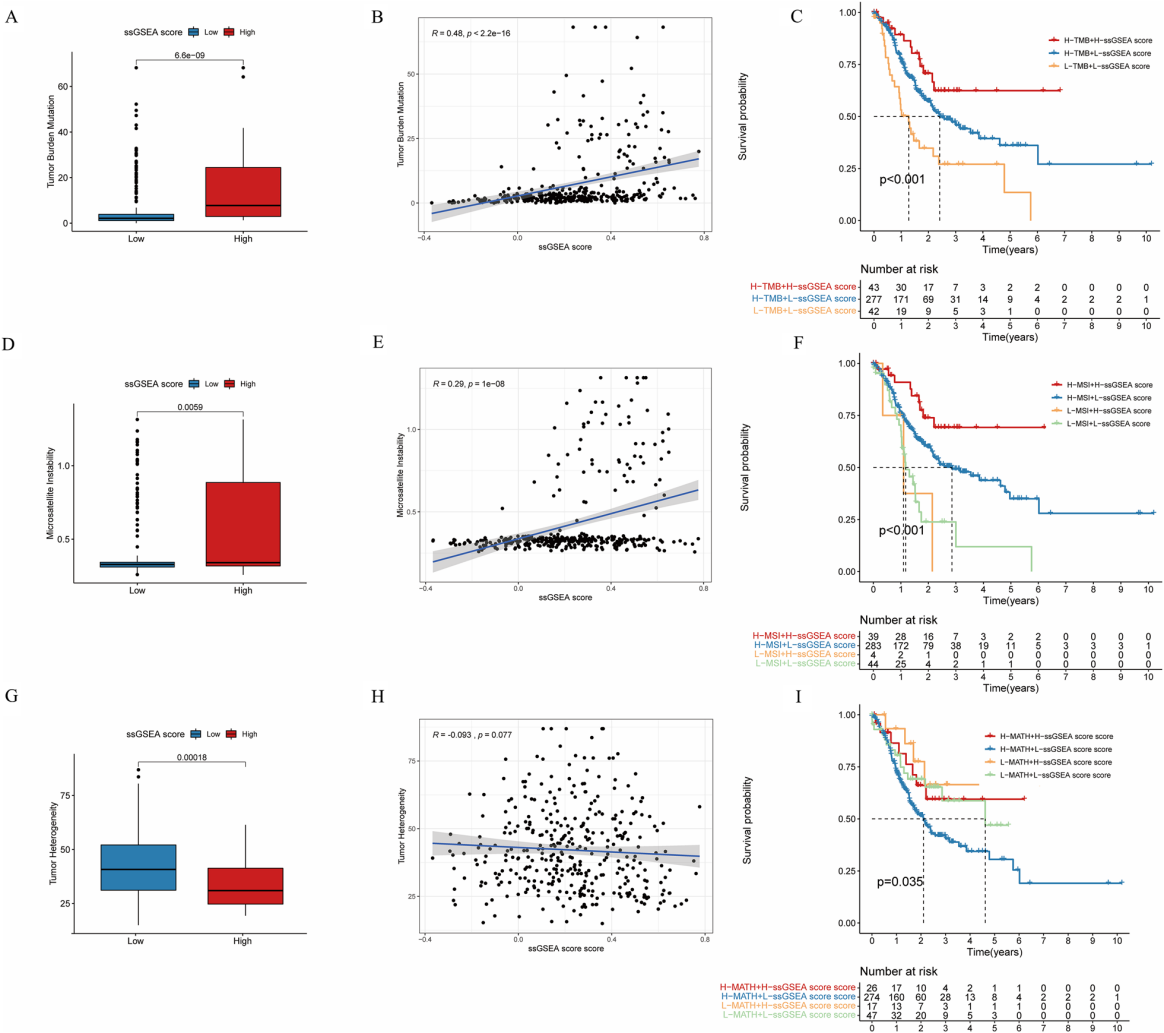
The correlations between immune response ssGSEA score and TMB, MSI and MATH scores. **A** The difference of TMB scores between GC patients with high and low immune response ssGSEA score; **B** The correlation between immune response ssGSEA score and TMB score; **C** OS of patients with different TMB score and immune response ssGSEA score; **D** The difference of MSI scores between GC patients with high and low immune response ssGSEA scores; **E** The correlation between immune response ssGSEA score and MSI score. **F** OS of patients with different MSI score and immune response ssGSEA score; **G** The difference of MATH score between GC patients with high and low immune response ssGSEA score; **H** The correlation between immune response ssGSEA score and MATH score; **I** OS of patients with different MATH score and immune response ssGSEA score

To further explore the relationship between immune response ssGSEA score and immunotherapy, we downloaded the genomic data of TCGA and sorted out the scores of TMB, MSI and MATH related to the efficacy of immunotherapy. In the immune index of TMB, we can see that the TMB score of the GC patients with higher immune response ssGSEA score was also significantly higher than that of the GC patients with lower scores (*P* < 0.001, Fig. 4A), indicating that the GC patients with higher scores had more gene mutations and had greater potential for immunotherapy. At the same time, the immune response ssGSEA score of GC patients was positively correlated with the TMB score (*R* = 0.48, *P* < 0.001, Fig. 4B). Among the GC patients analyzed in two subgroups, we found that the GC patients with high TMB score and high immune response ssGSEA score had the best prognosis. On the contrary, the GC patients with lower TMB and lower immune response ssGSEA score had worse prognosis (*P* < 0.001, Fig. 4C). Among the immunotherapy indexes of MSI, the GC patients with higher immune response ssGSEA score had a higher MSI score (*P* = 0.0059, Fig. 4D). At the same time, the immune response ssGSEA score was positively correlated with the MSI score (*R* = 0.29, *P* < 0.001, Fig. 4E), which further indicated that the immune response ssGSEA score was consistent with the related indexes of clinical efficacy of immunotherapy. At the same time, in the double subgroup analysis, the GC patients with high MSI score and high immune response ssGSEA score had the best prognosis, whereas the GC patients with low MSI score and low immune response ssGSEA score had the worst prognosis (*P* < 0.001, Fig. 4F). In terms of tumor heterogeneity, the GC patients with higher immune response ssGSEA score had lower MATH score (*P* < 0.001, Fig. 4G), indicating that these patients were not prone to immune tolerance and have a better effect on immunotherapy; at the same time, the immune response ssGSEA score was negatively correlated with the MATH score (*R* = − 0.093) (*R* = − 0.093, *P* = 0.077, Fig. 4H), but with no statistical difference (*P* = 0.077), which may be due to the limited sample size. In the two-subgroup analysis, the prognosis of GC patients with low MATH score and high immune response ssGSEA score was better, on the contrary, the prognosis of GC patients with high MATH score and low immune response ssGSEA score was the worst (*P* = 0.035, Fig. 4I).

### Prediction of survival probability of GC patients by nomogram

**Fig. 5:**
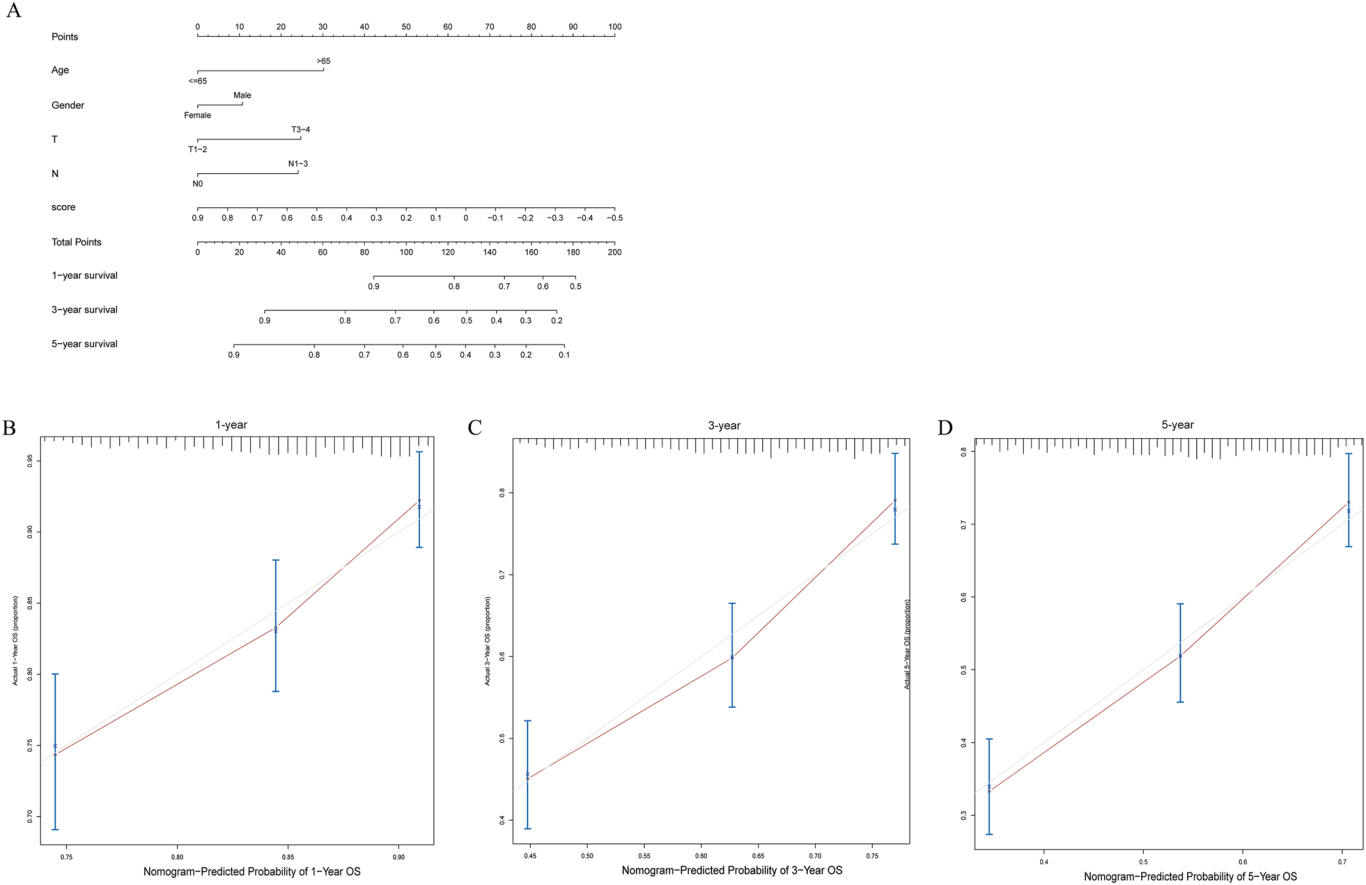
The construction Nomogram and the calibration curves. **A** Line chart (including age, sex, T and N stages, risk score); **B**–**D** Calibration curves for GC patients with OS of 1-year, 3-year and 5-year

To evaluate the survival probability of each GC patient, we used GC cohort from TCGA and GSE84437 from GEO to construct a line chart model to predict the survival of GC patients. Multivariate Cox regression analysis was used to screen variables with significant prognosis and clinical significance, including age, sex, T stage, N stage and model score. The detailed score of each variable is shown in the line chart (Fig. 5A). By calculating the score of each variable and calculating the total score, we can predict 1 year, 3 year and 5 year OS of GC patients. At the same time, the calibration curve was used to evaluate the prediction performance of Nomogram, and our prediction calibration curve was close to the standard curve (Fig. 5B–D), indicating that the prediction ability of the line chart was better.

### *COX7A1* screened by WGCNA and cytoscape analysis

**Fig. 6:**
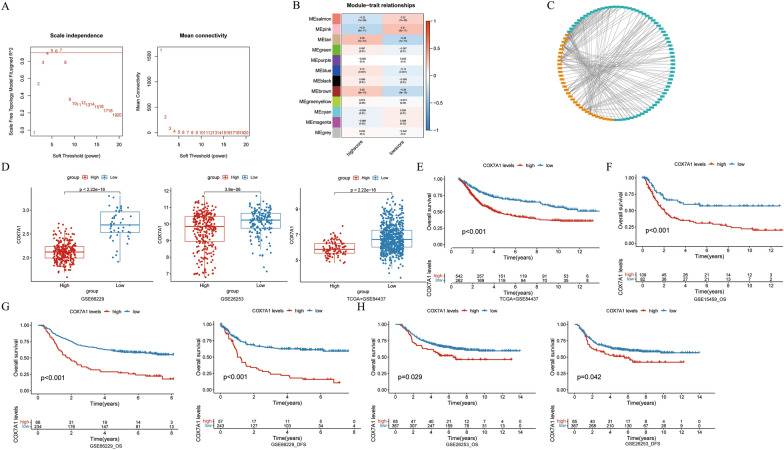
Construction of gene co-expression network and screening of *COX7A1*. **A** The network topology analysis of all kinds of soft threshold used to check the scale-free topology (the adjacency matrix was defined using the soft threshold of β = 4); **B** The correlation analysis between gene set module and immune response ssGSEA score; **C** The network map of the correlation between the SEs and the screened gene drawn by Cytoscape; **D** The differential expression of *COX7A1* between GC patients with high and low immune response ssGSEA score; **E** The difference of OS between groups of high and low expression of *COX7A1* in TCGA + GSE8443; **F** The difference of OS between groups of high and low expression of *COX7A1* in GSE15459; **G** The difference of OS and DFS between groups of high and low expression of *COX7A1* in GSE84437; **H** The difference of OS and DFS between groups of high and low expression of *COX7A1* in GSE262537

To further screen the molecular markers related to immunotherapy of GC, we used the GC data set from TCGA and GSE84437 from GEO for WGCNA. And *β* = 4 was selected as the best soft threshold to ensure that the network was scale-free (Fig. 6A). The correlation between the expression of gene set and the classification of gene model related to prognosis of immunotherapy was calculated. The genes under the pink module have the most significant expression correlation in the high and low score groups (| *R* |= 0.31, Fig. 6B). Therefore, the genes in the pink module were selected for further screening of molecular markers related to immunotherapy, and we used the genes in the pink module to intersect with the previously obtained differential genes, and finally obtained 835 meaningful genes for further screening. As the super-enhancer plays a more and more important role in the occurrence, development and treatment of tumor, we downloaded the super-enhancer gene set from the SEdb website and filter the part belonging to the stomach, and finally obtained 13,218 gastric SEs, and then the corresponding gene expression was extracted from our transcriptome data set. Then, to explore the expression correlation between the SEs and our screened gene set, we analyzed the correlation and set the filtering standard as the absolute value of the correlation coefficient greater than 0.8 (*P* < 0.0001). Finally, 31 SEs were obtained which were highly related to the expression of our screened gene set (Fig. 6C).

### The correlation between *COX7A1* and immune response ssGSEA score and its survival analysis

Based on our review of the literature, we selected *COX7A1* for further analysis due to its high expression in the gene set of interest. *COX7A1*, a key component of cytochrome oxidase, plays a crucial role in the energy metabolism of tumor cells, making it our focus of study. We found that the lower immune response ssGSEA score, the higher the expression of *COX7A1*, and there was a significant statistical difference between them, which was verified in three data sets, namely GSE66229, GSE26253 and TCGA + GSE84437 (all *P* < 0.001, Fig. 6D), showing the reliability of further screening molecular markers through our immune response ssGSEA score. To verify whether the *COX7A1* expression has the same effect on the OS and DFS of GC patients as our immune response ssGSEA score, we performed survival analysis of *COX7A1*. As we expected, in TCGA + GSE84437 and GSE15459 datasets, the OS of GC patients with high expression of *COX7A1* was significantly worse than those with low expression of *COX7A1* (both *P* < 0.001, Fig. 6E, F). In GSE66229 and GSE26253 data sets, the OS and DFS of GC patients with high expression of *COX7A1* were worse than those with low expression of *COX7A1* (all *P* < 0.05, Fig. 6G, H).

### High expression of *COX7A1* leads to chemotherapy resistance of 5-Fu and oxaliplatin

**Fig. 7:**
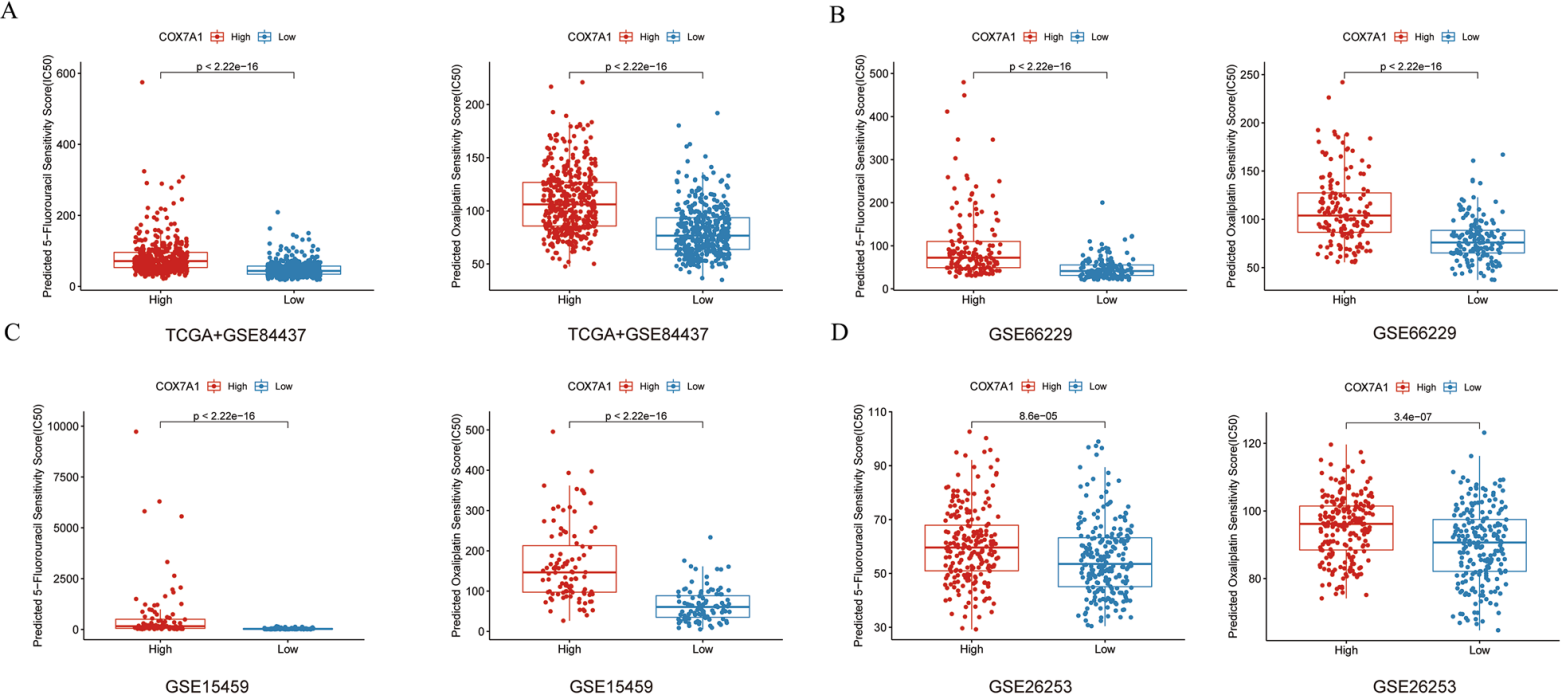
The correlation between the *COX7A1* expression and the drug sensitivity to 5-Fu and Oxaliplatin in **A** TCGA + GSE84437, **B** GSE66229, **C** GSE15459 and **D** GSE26253

Then, to further explore the correlation between the *COX7A1* expression and the drug sensitivity of commonly used chemotherapeutic drugs in GC patients, we calculated the drug sensitivity scores of TCGA + GSE84437, GSE26253, GSE15459 and GSE66229 using oncoPredict package, and reached a consistent conclusion, that is, in GC patients with high expression of *COX7A1*, the drug sensitivity of GC patients to 5-Fu and Oxaliplatin decreased; on the contrary, GC patients with low expression of *COX7A1*, drug sensitivity to 5-Fu and Oxaliplatin increased (all *P* < 0.001, Fig. 7A–D), suggesting that those patients with low expression of *COX7A1* can choose 5-Fu or Oxaliplatin-based chemotherapy, while others should avoid using these two drugs with little clinical efficacy.

### *COX7A1* regulates tumor infiltration of immune cells and then affects the immune microenvironment

**Fig. 8:**
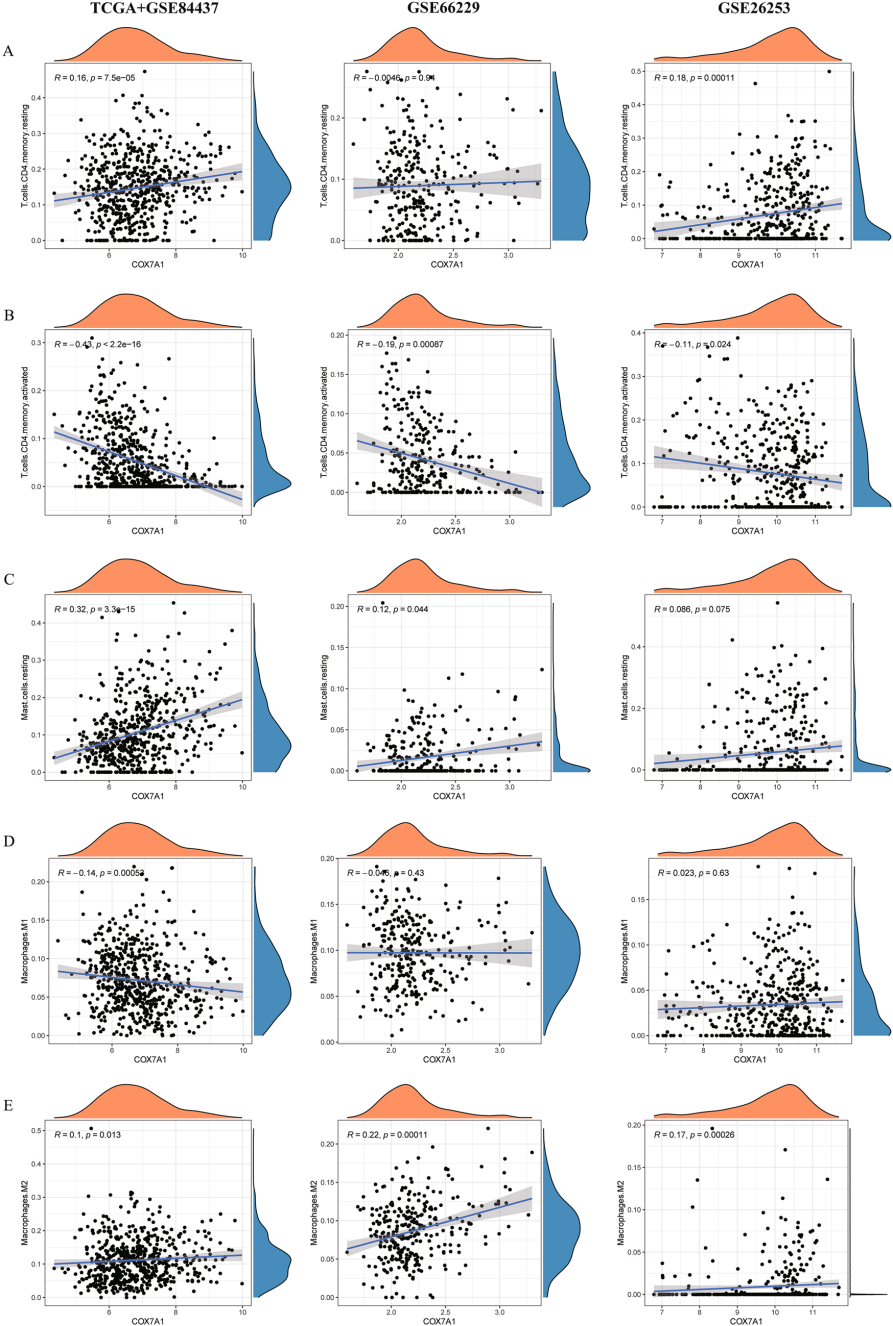
Analysis of the correlation between the *COX7A1* expression and the content of tumor infiltrating immune cells. The correlation between the *COX7A1* expression and the content of **A** resting CD4^+^ memory T cells, **B** activated CD4^+^ memory T cells, **C** resting mast cells, **D** M1 macrophages and **E** M2 macrophages

To further explore the relationship between *COX7A1* and immunotherapy in GC patients, we used cibersort deconvolution algorithm to calculate the content of 22 kinds of immune cells in GC patients with TCGA + GSE84437, GSE66229 and GSE26253 data sets, and calculated the correlation between the expression of *COX7A1* and the content of these immune cells. We found that in the three data sets, the *COX7A1* expression was negatively correlated with the content of activated memory CD4^+^ T cells and positively correlated with the content of resting memory CD4^+^ T cells and resting mast cells (partly *P* < 0.05, Fig. 8A–C). At the same time, the *COX7A1* expression was negatively correlated with the content of M1 macrophages (*P* = 0.006), and positively correlated with the content of M2 macrophages (partly *P* < 0.05, Fig. 8D, E). This may explain the poor prognosis of patients with high expression of *COX7A1* from the perspective of macrophages, which needs to be further verified in basic cell experiments. To further explore the immune microenvironment of GC patients, we additionally applied the ESTIMATE algorithm to calculate the Immune Score and Stromal Score, and it was found that those patients with high expression of *COX7A1* were associated with higher Immune Score and Stromal Score, indicating their poor prognosis (Additional file 5: Fig. S2). Additionally, based on single-cell data, it is found that the *COX7A1* expression was positively correlated with the content of Fibroblasts and Endothelial cells (Additional file 5: Fig. S3).

### Low expression of *COX7A1* in tumor tissues may promote the invasion and metastasis

**Fig. 9:**
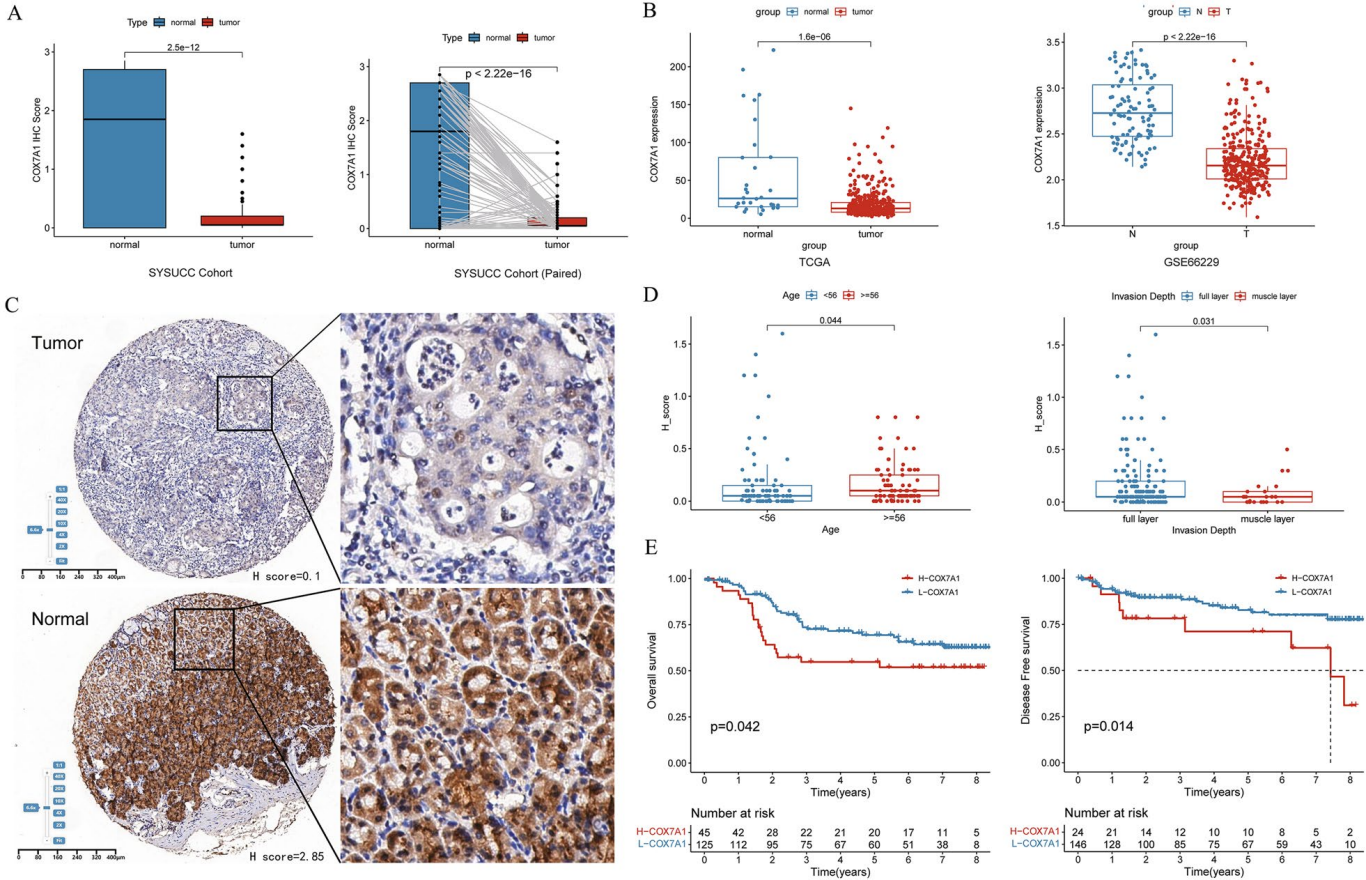
The Correlation between differential expression of *COX7A1* and clinical information. **A** The differential expression of *COX7A1* between tumor tissues and normal tissues of GC patients; **B** The *COX7A1* expression in tumor tissues and normal tissues in TCGA and GSE66229; **C** Immunohistochemical map of tumor tissues and normal tissues of GC patients (5 × microscope); **D** The correlation between *COX7A1* expression with the age and the depth of pathological invasion in GC patients; **E** The correlation between *COX7A1* expression with OS and DFS in GC patients

To further explore the role of *COX7A1* gene in gastric cancer, pathological sections and immunohistochemical staining of tumor and paracancerous area were performed in 170 GC patients in Sun Yat-sen University Cancer Center. The clinical information of the patients is detailed in Table 2 (SYSUCC Cohort). Through the analysis of immunohistochemical data, we found that the expression of *COX7A1* in tumor tissue was significantly lower than that in paracancerous tissue (both *P* < 0.001, Fig. 9A). At the same time, we also reached a consistent conclusion in public data in TCGA and GSE66229 (both *P* < 0.001, Fig. 9B), that is, *COX7A1* was significantly lower in tumor tissues than in paracancerous and normal tissues. And through the immunohistochemical image, *COX7A1* protein was mainly concentrated in the normal glandular area, and the expression was very low in the cancerous tumor glands (Fig. 9C). Then, in the analysis of *COX7A1* and clinical information, we found that the expression of *COX7A1* was higher in elderly patients (over 56 years old) and gastric cancer patients whose tumors invaded the whole layer of gastric wall (both *P* < 0.05, Fig. 9D), indicating that *COX7A1* may be related to the invasion and metastasis of gastric cancer patients. On the other hand, there was no significant difference between *COX7A1* and vascular tumor thrombus, lymph node metastasis, nerve invasion, differentiation and staging (Additional file 5: Fig. S4A–E). Then, using the follow-up data, we found that GC patients with higher expression of *COX7A1* had shorter OS and DFS (both *P* < 0.05, Fig. 9E) than patients with low expression. To further explore stage-specific patterns, we additionally investigated the influence of *COX7A1* on prognosis by TNM stage (Additional file 5: Fig. S5), showing acceptable prediction value. Due to the obviously limited number of patients in stage I, we grouped stage I and II together, and stage III and IV together. Furthermore, among these patients, through multivariable Cox regression analysis, *COX7A1* was considered to be an independent prognostic factor for OS and DFS (both *P* < 0.05, Additional file 4: Table S4). Finally, in our Immune Cohort, those GC patients with higher expression of *COX7A1* had shorter OS and DFS (Additional file 5: Fig. S6); and *COX7A1* in patients with TRG 2 or 3 was higher than that in patients with TRG 0 or 1 (*P* = 0.073, Wilcox. test) (Additional file 5: Fig. S4F), but there was no significant difference between the two groups, which was considered to be the reason for the insufficient sample size.

**Table 2:** Baseline characteristics of the 170 GC patients with immunohistochemistry data from Sun Yat-sen University Cancer Center

SYSUCC Cohort	H score_high (*N* = 76)	H score_low (*N* = 94)	*P*-value
Age			0.211
< 56	33 (43.4%)	51 (54.3%)	
> = 56	43 (56.6%)	43 (45.7%)	
Stage			0.121
I + II	52 (68.5%)	54 (57.4%)	
III + IV	22 (28.9%)	40 (42.6%)	
NA	2 (2.6%)	0 (0%)	
Pathological type			0.096
Moderately differentiated	21 (27.6%)	15 (16.0%)	
Poorly differentiated	55 (72.4%)	79 (84.0%)	
Invasion depth			0.21
Muscle layer and above	7 (9.2%)	16 (17.0%)	
Full layer	69 (90.8%)	78 (83.0%)	
Lymph metastasis			0.889
No	25 (32.9%)	33 (35.1%)	
Yes	51 (67.1%)	61 (64.9%)	
Nerve invasion			0.86
No	20 (26.3%)	27 (28.7%)	
Yes	56 (73.7%)	67 (71.3%)	
Vascular tumor thrombus			0.647
No	32 (42.1%)	44 (46.8%)	
Yes	44 (57.9%)	50 (53.2%)	

*NA* not available

## Discussion

GC is one of the most common malignant tumors in the world [43]. The latest statistics on the disease show that GC is currently listed as the second largest cause of cancer-related death in the world [44, 45]. Although GC has improved in recent years, the prognosis of GC is still poor because of large tumor heterogeneity, limited treatment, low early diagnosis rate and so on [46]. In recent years, with the application of sequencing technology, many studies have shown that driving gene mutations and molecular pathological typing affect the prognosis of cancer. In addition, due to the lack of novelty and rich validation, the existing clinical prognostic models have not been widely accepted. At the same time, even though breakthroughs have been made in the diagnosis and treatment of GC in the past few years, surgery is still the main treatment option for GC patients. And because a large number of patients were initially diagnosed in late stage, the prognosis of GC patients is still poor. Therefore, there is an urgent need to distinguish high-risk GC patients and identify possible molecular targets for the benefit of GC patients.

Immunotherapy broke the traditional concept of surgical treatment, chemotherapy and targeted therapy in the treatment of GC, and significantly improved the survival rate of some patients [47]. However, even so, only less than 25% of GC patients can benefit [48]. Therefore, it is an important clinical problem to find biomarkers that can accurately predict the response to immunotherapy, to formulate corresponding individualized treatment plans, and to prevent injuries to patients caused by excessive and inappropriate treatment. At present, biomarkers used to predict the efficacy of PD1/PD-L1 monoclonal antibodies include combined positive score (CPS) [15], MSI [49] and TMB [50]. However, all these biomarkers focus on the inherent characteristics of the tumor, thus ignoring the assessment of the tumor microenvironment on which tumor growth depends, resulting in the lack of effectiveness of its prediction.

In our study, we downloaded data sets related to GC from TCGA, GEO and clinical trials of immunotherapy for GC. According to these data sets, we selected 455 prognosis-related genes that were different in the effectiveness of immunotherapy, and then continued ssGSEA analysis and took the difference between the two gene sets to get the immune response ssGSEA score to build the model. The prognostic effect of the immune response ssGSEA score obtained by the immunotherapy prognosis-related gene model was effectively verified in the training set of TCGA + GSE84437 and the verification set cohort of GSE66229, GSE26253 and GSE15459. To expand the clinical use of the model, we generated a new line chart, including the clinicopathological characteristics and model risk score of GC patients. The calibration curve also proves that our line chart has a good linear fit for predicting prognosis. Then, we also evaluated the correlation between the model score and the commonly used molecular markers for predicting the efficacy of immunotherapy for GC, such as TMB, MSI and MATH. The results show that our model score is consistent with the effect of these markers on the prognosis of GC patients, which also provides a theoretical basis for the clinical application of immune response ssGSEA score. Then, we use the established model for further molecular screening, using WGCNA to construct gene co-expression network, cytoscape software to analyze and select the corresponding modules, and to search the corresponding literature for the selected genes, and finally select *COX7A1* as the molecule for further research. We first evaluated the relationship between the expression of *COX7A1* and our model, and found that the lower the expression of *COX7A1* in GC patients with higher immune response ssGSEA score, indicating that *COX7A1* may be a poor prognostic factor; then we analyzed the prognosis of *COX7A1*, and proved that *COX7A1* was a poor prognostic factor in multiple data sets, while patients with high expression of *COX7A1* were resistant to 5-Fu and oxaliplatin. At the same time, we explored the immune mechanism of *COX7A1* affecting the prognosis of GC patients. According to the analysis of multiple data sets based on Cibersort deconvolution algorithm, *COX7A1* may affect the immune microenvironment of GC patients by up-regulating resting CD4^+^ T cells, thus affecting the prognosis of GC patients [51, 52]. But what is puzzling is that *COX7A1* can also up-regulate the increase the content of CD8^+^ T cells, which may be due to the fact that *COX7A1* can up-regulate the expression of some immunosuppressive molecules or immunosuppressive cells such as Treg, thus interfering with the normal anti-tumor effect of CD8^+^ T cells, so it also creates conditions for the treatment of PD1/PDL1. If it can relieve the immunosuppressive state of high expression of *COX7A1*, it can better play the role of CD8^+^ T cells in killing tumor cells. This part is worthy of our further verification in the following experiments [53, 54]. Finally, we explored the clinical application of *COX7A1*. We found that the expression of *COX7A1* in tumor tissues was significantly lower than that in paracancerous/normal gastric tissues in both TCGA + GSE84437 and GSE66229 cohorts of GC, which was also verified in the tissue microarray of clinical patients in our hospital. We also explored the clinical value of *COX7A1* in GC. In the correlation analysis of clinical data, we found that the higher the expression of *COX7A1* in GC patients whose pathological sections invaded the whole layer, which suggested that *COX7A1* may be a protein molecule related to invasion and metastasis, which needs further verification in cytological experiments.

The role of COX subunits in many cancers has also been studied [55–57]. For example, Mishra et al. used the available microarray database to compare the expression of different COX subunit genes in human lung adenocarcinoma tissue and normal lung tissue. The results showed that the expression of *COX7A1* in cancer tissue was much lower than that in normal lung tissue, suggesting that *COX7A1* may inhibit the occurrence and development of lung cancer [55]. At the same time, *COX7A1* was found to be able to inhibit lung cancer cell proliferation and colony formation and promote apoptosis [24]. In addition, the overexpression of *COX7A1* blocks autophagy by down-regulation of PGC-1α and up-regulation of NOX2 [24]. Further analysis shows that the effect of *COX7A1* on cell viability depends partly on the inhibition of autophagy [26]; and it was further revealed that overexpression of *COX7A1* could enhance the activity of complex IV in TCA cycle and mitochondrial electron transport chain, thus increasing the sensitivity of lung cancer cells to ferroptosis induced by cysteine deprivation [26]. On the other hand, *COX7A1* blocks autophagy flux and inhibits mitochondrial kinetics, mitochondrial biogenesis and mitochondrial autophagy, thus affecting the activity of complex I and II in mitochondrial electron transport chain [24]. *COX7A1* may up-regulate the expression of immunosuppression-related proteins through tumor-related pathways, thus forming an immunosuppressive microenvironment, which requires further cytological experiments to verify the potential mechanism of our differential response of *COX7A1* to immunotherapy in GC patients.

In this study, the higher the expression of *COX7A1* in GC tissue, the worse the prognosis; but on the other hand, the expression of *COX7A1* in tumor tissue is lower than that in normal tissue. The same biological model has also been reported in other tumor studies, for example, in breast cancer, the expression of *MAPT* in tumor tissues is significantly higher than that in normal glandular tissues, and the OS and DFS of breast cancer patients with high *MAPT* expression are significantly higher than those of tumor patients with low *MAPT* expression. The article explains that the possible reason is that the change of *MAPT* expression affects the sensitivity of breast cancer cells to chemotherapeutic drugs, which also has a certain guiding significance for our research. We can explore whether the change of *COX7A1* expression will change the sensitivity of chemotherapeutic drugs or immunotherapy for GC, which is worthy of further validation through studies with larger sample sizes and cytological experiments.

Previous studies have also found that M1 and M2 macrophages play an important role in the occurrence and development of tumors [58]. M2 macrophages can promote tumor growth and metastasis [59], but M1 macrophages can promote inflammation, leading to tumor-associated macrophage (TAM) infiltration, and then inhibit the vitality of tumor cells [60]. In our study, consistent with the above ideas, it is believed that *COX7A1* may promote M2 differentiation and inhibit M1 differentiation. Moreover, it is found that the *COX7A1* expression is positively correlated with the Immune Score, Stromal Score and the content of resting CD4^+^ memory T cells, and negatively correlated with the content of activated CD4^+^ memory T cells, indicating that *COX7A1* may inhibit the activation of CD4^+^ memory T cells to result in poor prognosis. Additionally, the *COX7A1* expression was preliminary found to be positively correlated with the content of Fibroblasts and Endothelial cells, indicating *COX7A1* may plays a role in chemotherapy resistance, which will serve as a breakthrough for our further research.

However, this study also has some limitations. First, bioinformatics research for this work is conducted partly on publicly available data sets. Second, we need to ensure that the results of this survey are accurate using clinical trial participants in a prospective study design. Second, although immunohistochemical experiments have been carried out to verify the results of model screening, large-scale protein sequencing analysis will be a better choice. Third, we analyzed the effects of the established model on MSI, TMB and tumor heterogeneity, but the underlying mechanism is still unclear. Therefore, to better understand the potential mechanism of the established model and screened *COX7A1* molecules on immunotherapy response to GC, further cytological and animal experimental studies in vivo and in vitro are needed.

## Conclusions

Our results show that the immune response ssGSEA score established based on genes of differential immunotherapy response can well distinguish the prognosis of GC patients, and the immune response ssGSEA score is in good agreement with the commonly used immune-related indexes (TMB, MSI and MATH). The *COX7A1* gene was obtained by further molecular screening using immune response ssGSEA score, and the predictive effect of *COX7A1* on the prognosis of GC patients was verified in several data sets: GC patients with high expression of *COX7A1* in GC tissues had poor prognosis; the *COX7A1* expression in the tumor tissues was significantly lower than that in the paracancerous/normal tissues; and the *COX7A1* expressions were higher in patients with older age and deeper invasion. In terms of drug sensitivity, GC patients with low expression of *COX7A1* were more sensitive to 5-Fu and Oxaliplatin. Tumor infiltration immune cell analysis for *COX7A1* showed that *COX7A1* may affect the clinical efficacy of immunotherapy by promoting M2 macrophages differentiation and inhibiting M1 macrophages differentiation.

### Supplementary Information

**Additional file 1: Table S1.** Characteristics of the TCGA and GEO datasets used in the study.

**Additional file 2: Table S2.** Baseline characteristics and TRG evaluation of the 24 GC patients in the Immune Cohort.

**Additional file 3: Table S3.** Gene list and KEGG enrichment analysis of the protective genes (47 genes) and risky genes (408 genes).

**Additional file 4: Table S4.** Multivariable Cox regression analysis showed COX7A1 was an independent prognostic factor for OS and DFS in the SYSUCC Cohort.

**Additional file 5: Fig S1.** Survival analysis (OS and DFS) between high and low score ssGSEA groups of GC patients at different TNM stages based on public datasets. **Fig S2.** Immune Score and Stromal Score by ESTIMATE algorithm between high and low COX7A1 expression groups. **Fig S3.** Differential COX7A1 expression in different cell types based on single-cell data. **Fig S4.** The correlations between COX7A1 expression and clinical information. (A) The correlation between COX7A1 expression and vascular tumor thrombus; (B) The correlation between COX7A1 expression and lymph node metastasis; (C) The correlation between COX7A1 expression and nerve invasion; (D) The correlation between COX7A1 expression and pathological differentiation; (E) The correlation between COX7A1 expression and the tumor stage of GC patients; (F) The correlation between different response TRG and COX7A1 in GC patients treated with immunotherapy. **Fig S5.** Survival analysis (OS and DFS) between high and low COX7A1 expression groups of GC patients at different TNM stages in the SYSUCC Cohort. **Fig S6.** Survival analysis (OS and DFS) between high and low COX7A1 expression groups of GC patients in the Immune Cohort.

**Additional file 6.** Supplementary Methods.

## Data Availability

All data generated or analyzed during this study are included in this published article and its Additional files.

## Abbreviations

GC: Gastric cancer
TME: Tumor microenvironment
GEO: Gene Expression Omnibus
TCGA: The Cancer Genome Atlas
ssGSEA: Single Sample Gene Set Enrichment Analysis
TMB: Tumor mutation burden
MSI: Microsatellite instability
MATH: Mutant-allele tumor heterogeneity
WGCNA: Weighted Correlation Network Analysis
OS: Overall survival
DFS: Disease-free survival
ICIs: Immune checkpoint inhibitors
NAL: Neoantigen load
CAN: Copy alternation number
dMMR: Mismatch repair deficiency
SE: Super enhancer
TE: Typical enhancer
GSVA: Gene set variation analysis
CDF: Cumulative distribution function
KEGG: Kyoto Encyclopedia of Genes and Genomes
TOM: Topology measurement
GDSC: Genomics of Drug Sensitivity in Cancer
IC_50_: Half maximal inhibitory concentration
TIIC: Tumor Infiltration Immune Cell
SVR: Support Vector Regression
TRG: Tumor regression grade
PFS: Progression-free survival
CPS: Combined positive score
TAM: Tumor-associated macrophage
