# Supplementary material for: Construction of a gene model related to the prognosis of patients with gastric cancer receiving immunotherapy and exploration of COX7A1 gene function
Source: Eur J Med Res. 2024 Mar 17;29:180. doi: 10.1186/s40001-024-01783-x (PMC11337786; doi:10.1186/s40001-024-01783-x)
Supplement: Supplementary file 1 — Additional file 1: Table S1. Characteristics of the TCGA and GEO datasets used in the study. [file 40001_2024_1783_MOESM1_ESM.docx]

| **Dataset** | **Platform** | **Source** | **Normal tissues** | **Tumor tissues** |
| --- | --- | --- | --- | --- |
| TCGA  GSE84437 | RNA-Seq  Illumina HumanHT-12 V3.0 expression beadchip | Stomach  Stomach | 32  0 | 375  433 |
| GSE66229 | Affymetrix Human Genome U133 Plus 2.0 Array | Stomach | 100 | 300 |
| GSE15459 | Affymetrix Human Genome U133 Plus 2.0 Array | Stomach | 0 | 191 |
| GSE26253  GSE26942  Kim Cohort | Illumina HumanRef-8 WG-DASL v3.0  Illumina HumanHT-12 V3.0 expression beadchip  RNA-Seq | Stomach  Stomach  Stomach | 0  12  0 | 432  202  55 |
