# Supplementary material for: Construction of a gene model related to the prognosis of patients with gastric cancer receiving immunotherapy and exploration of COX7A1 gene function
Source: Eur J Med Res. 2024 Mar 17;29:180. doi: 10.1186/s40001-024-01783-x (PMC11337786; doi:10.1186/s40001-024-01783-x)
Supplement: Supplementary file 2 — Additional file 2: Table S2. Baseline characteristics and TRG evaluation of the 24 GC patients in the Immune Cohort. [file 40001_2024_1783_MOESM2_ESM.docx]

| **Immune Cohort** | **N=24** |
| --- | --- |
| **Survival status** | |
| Alive | 17 (70.8%) |
| Dead | 7 (29.2%) |
| **Recurrence status** | |
| No Recurrence | 8 (33.3%) |
| Recurrence | 16 (66.7%) |
| **Gender** | |
| Female | 9 (37.5%) |
| Male | 15 (62.5%) |
| **Age** | |
| < 56 | 12 (50.0%) |
| >= 56 | 12 (50.0%) |
| **Stage** | |
| I | 3 (12.5%) |
| II | 6 (25.0%) |
| III | 5 (20.8%) |
| IV | 10 (41.7%) |
| **Lauren classification** | |
| Diffuse | 8 (33.3%) |
| Intestinal | 9 (37.5%) |
| Mixed | 6 (25.0%) |
| NA | 1 (4.2%) |
| **Stage T** | |
| T1 | 1 (4.2%) |
| T2 | 5 (20.8%) |
| T3 | 12 (50.0%) |
| T4 | 6 (25.0%) |
| **Stage N** | |
| N0 | 8 (33.3%) |
| N1 | 3 (12.5%) |
| N2 | 6 (25.0%) |
| N3 | 7 (29.2%) |
| **Stage M** | |
| M0 | 15 (62.5%) |
| M1 | 9 (37.5%) |
| **TRG grade** | |
| 0 | 1 (94.2%) |
| 1 | 2 (8.3%) |
| 2 | 14 (58.3%) |
| 3 | 7 (29.2%) |

**TRG:** tumor regression grade.
