# Supplementary material for: Construction of a gene model related to the prognosis of patients with gastric cancer receiving immunotherapy and exploration of COX7A1 gene function
Source: Eur J Med Res. 2024 Mar 17;29:180. doi: 10.1186/s40001-024-01783-x (PMC11337786; doi:10.1186/s40001-024-01783-x)
Supplement: Supplementary file 5 — Additional file 5: Fig S1. Survival analysis (OS and DFS) between high and low score ssGSEA groups of GC patients at different TNM stages based on public datasets. Fig S2. Immune Score and Stromal Score by ESTIMATE algorithm between high and low COX7A1 expression groups. Fig S3. Differential COX7A1 expression in different cell types based on single-cell data. Fig S4. The correlations between COX7A1 expression and clinical information. (A) The correlation between COX7A1 expression and vascular tumor thrombus; (B) The correlation between COX7A1 expression and lymph node metastasis; (C) The correlation between COX7A1 expression and nerve invasion; (D) The correlation between COX7A1 expression and pathological differentiation; (E) The correlation between COX7A1 expression and the tumor stage of GC patients; (F) The correlation between different response TRG and COX7A1 in GC patients treated with immunotherapy. Fig S5. Survival analysis (OS and DFS) between high and low COX7A1 expression groups of GC patients at different TNM stages in the SYSUCC Cohort. Fig S6. Survival analysis (OS and DFS) between high and low COX7A1 expression groups of GC patients in the Immune Cohort. [file 40001_2024_1783_MOESM5_ESM.docx]

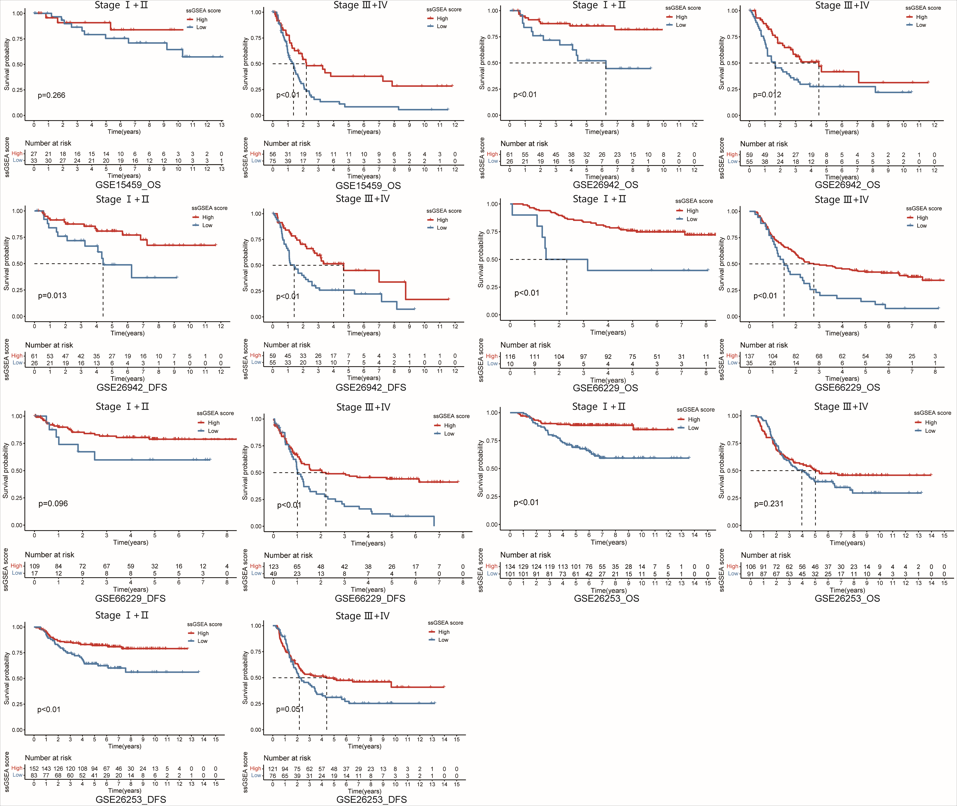


**Supplementary Fig. 1.** Survival analysis (OS and DFS) between high and low score ssGSEA groups of GC patients at different TNM stages based on public datasets.


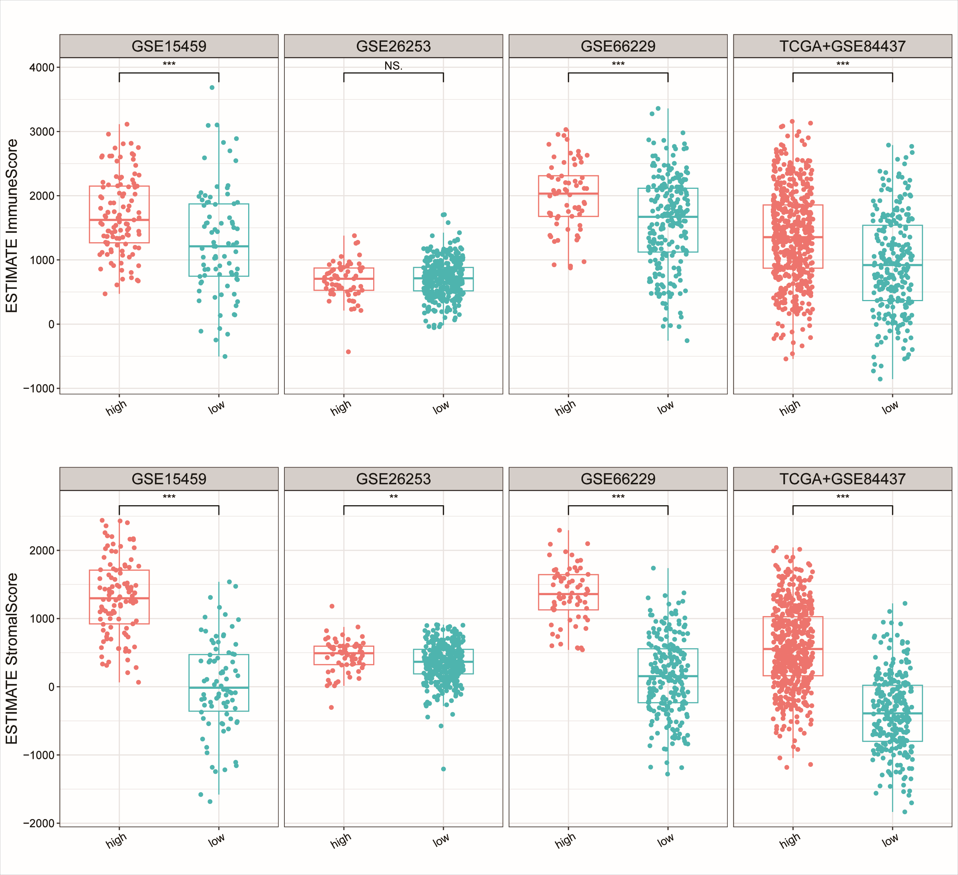


**Supplementary Fig. 2.** Immune Score and Stromal Score by ESTIMATE algorithm between high and low *COX7A1* expression groups.


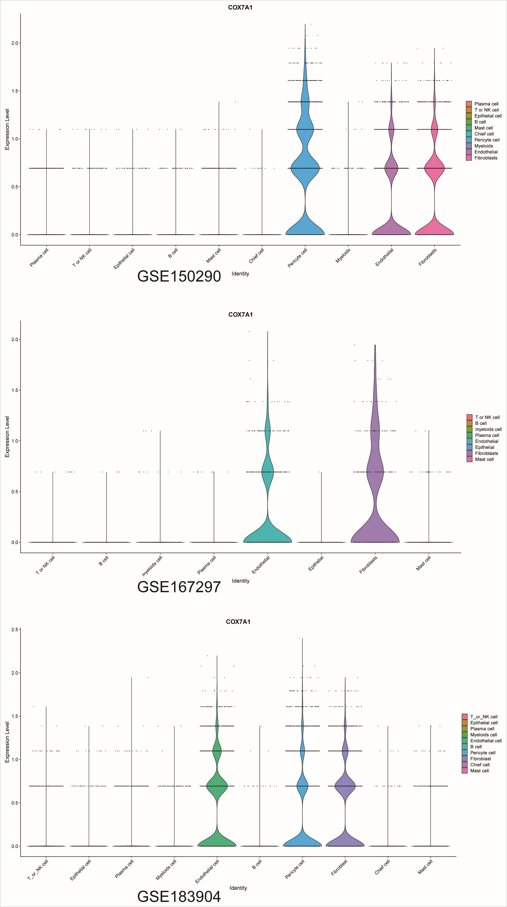


**Supplementary Fig. 3.** Differential *COX7A1* expression in different cell types based on single-cell data.


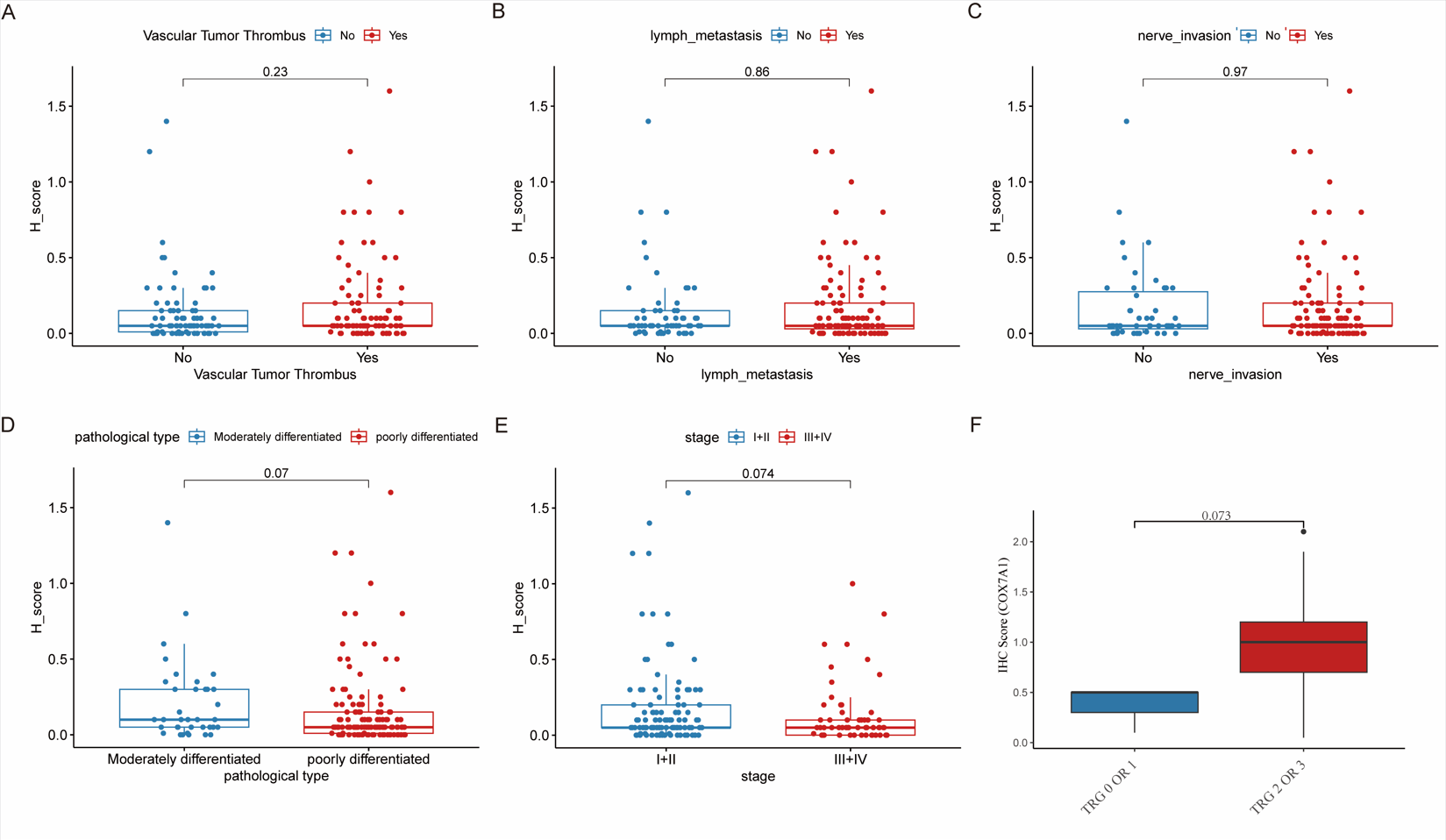


**Supplementary Fig. 4.** The correlations between *COX7A1* expression and clinical information. **(A)** The correlation between *COX7A1* expression and vascular tumor thrombus; **(B)** The correlation between *COX7A1* expression and lymph node metastasis; **(C)** The correlation between *COX7A1* expression and nerve invasion; **(D)** The correlation between *COX7A1* expression and pathological differentiation; **(E)** The correlation between *COX7A1* expression and the tumor stage of GC patients; **(F)** The correlation between different response TRG and *COX7A1* in GC patients treated with immunotherapy.


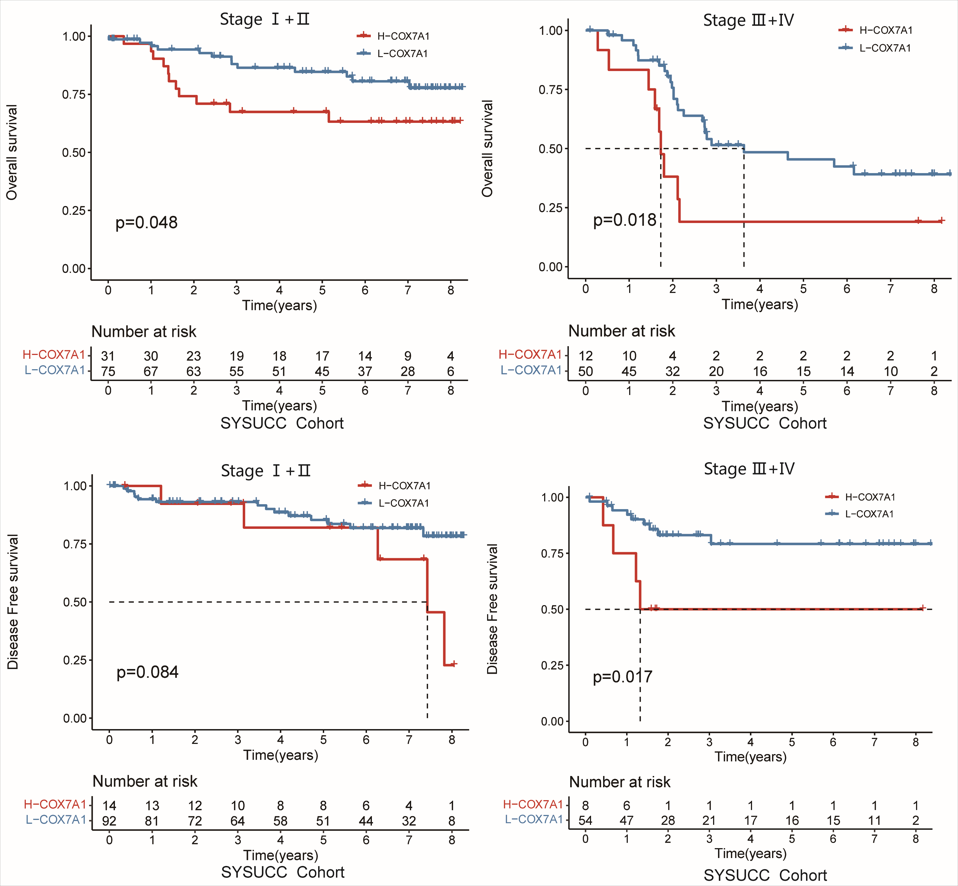


**Supplementary Fig. 5.** Survival analysis (OS and DFS) between high and low *COX7A1* expression groups of GC patients at different TNM stages in the SYSUCC Cohort.


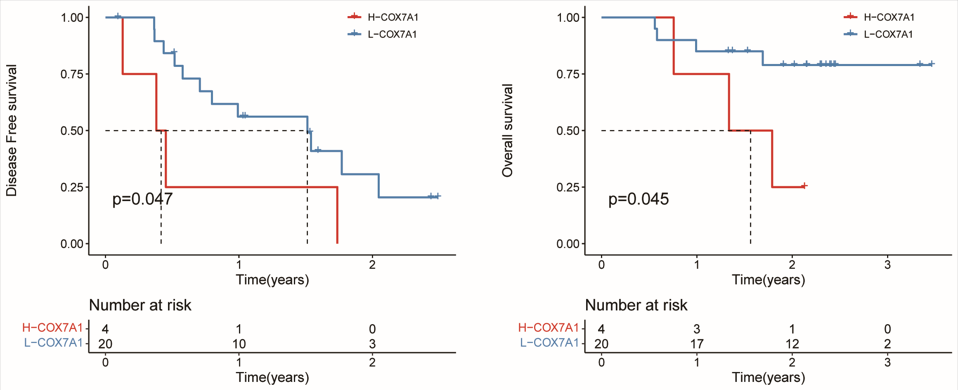


**Supplementary Fig. 6.** Survival analysis (OS and DFS) between high and low *COX7A1* expression groups of GC patients in the Immune Cohort.
