# Supplementary material for: Construction of a gene model related to the prognosis of patients with gastric cancer receiving immunotherapy and exploration of COX7A1 gene function
Source: Eur J Med Res. 2024 Mar 17;29:180. doi: 10.1186/s40001-024-01783-x (PMC11337786; doi:10.1186/s40001-024-01783-x)
Supplement: Supplementary file 6 — Additional file 6. Supplementary Methods. [file 40001_2024_1783_MOESM6_ESM.docx]

Supplementary Methods

1 Single-cell Data Download and Processing

For single-cell data, the following single-cell data sets were downloaded from the GEO website (<https://www.ncbi.nlm.nih.gov/geo/>), including GSE150290, GSE167297 and GSE183904. Those cells with nFeature_RNA <200 or >8,000, mitochondrial sequencing count >20% and nCount_RNA >15,000 were excluded, and then transitional cells or double cells were filtered out using the "Doubletfinder" package[[1](#_ENREF_1)], and the parameters were set to default. The batch effect of normalized and sequenced data was accomplished by using "seurat" and "harmony" packages respectively. Next, "FindVariableFeatures" package was used to calculate highly variable genes and the output result was used for principal component analysis (PCA). The first 20 principal components (PCs) were selected for subsequent dimension-reducing analysis. Then the cell markers reported in the literature were summarized and the characteristic markers were selected to determine the cell types.

2 Tumor Microenvironment Analysis

To explore the immune microenvironment of GC patients, we applied the ESTIMATE algorithm in “estimate” and “limma” package of R Software to calculate the Immune Score and Stromal Score. The Estimate Score was used to describe tumor purity. This analysis was based on the gene expression data retrieved from the following datasets: TCGA, GSE84437, GSE15459, GSE26253 and GSE66229.

[1] Mcginnis C S, Murrow L M, Gartner Z J. DoubletFinder: Doublet Detection in Single-Cell RNA Sequencing Data Using Artificial Nearest Neighbors[J]. Cell Syst, 2019, 8(4): 329-337 e4.
